# A Model for the Fate of a Gas Bubble Interacting with a Wire Mesh

**DOI:** 10.1021/acs.iecr.3c00265

**Published:** 2023-05-08

**Authors:** Rahul Subburaj, Yali Tang, Niels G. Deen

**Affiliations:** † Power and Flow Group, Department of Mechanical engineering, Eindhoven University of Technology, P.O. Box 513, 5600 MB Eindhoven, The Netherlands; ‡ Eindhoven Institute for Renewable Energy Systems, Eindhoven University of Technology, P.O. Box 513, 5600 MB Eindhoven, The Netherlands

## Abstract

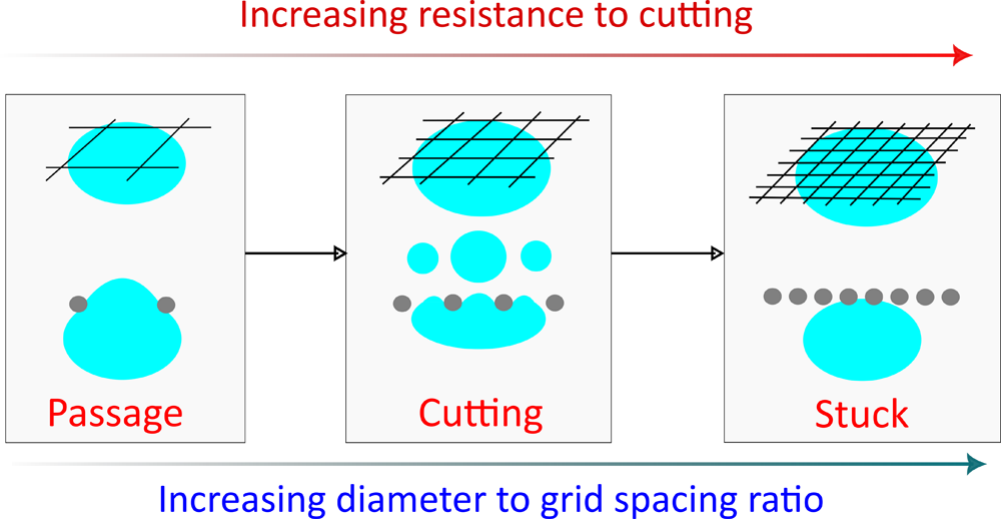

In the concept of a microstructured bubble column reactor, microstructuring of the catalyst carrier is realized by introducing a static mesh of thin wires coated with catalyst inside the column. Meanwhile, the wires also serve the purpose of cutting the bubbles, which in turn results in high interfacial area and enhanced interface hydrodynamics. However, there are no models that can predict the fate of bubbles (cut/stuck) passing through these wires, thus making the reactor optimization difficult. In this work, based on several typical bubble–wire interacting configurations, we analyze the outcomes by applying the energy balance of the bubble focusing on buoyancy and surface tension. Two limiting cases of viscosity, corresponding to the ability of the bubble to reconfigure into the lowest energy state, are investigated. Upon analysis, it is observed that a narrow mesh spacing and a smaller bubble Eötvös number generally result in bubbles getting stuck underneath the wire. We have obtained the threshold grid spacing and the critical Eötvös number for bubble passage and bubble cutting, which are verified by the direct numerical simulation results of bubble passing through a single mesh opening. The derived energy balance is generalized to large meshes with multiple openings and different configurations. Finally, a closure model based on the outcomes of energy-balance analysis is proposed for Euler–Lagrange simulations of microstructured bubble columns.

## Introduction

Bubble column reactors (BCRs) possess excellent heat and mass transfer capabilities making them popular in the refineries as well as chemical and pharmaceutical industries.^1^ They are easy to construct, require little maintenance, and have low operating costs. In a bubble column, the reaction output generally depends on hydrodynamic and mass transfer characteristics. In particular bubble hydrodynamics and size distribution play important roles. At real working conditions, i.e., higher superficial gas velocities, the bubbles can coalesce resulting in a lower specific interfacial area thus reducing the chemical conversion and yield. Youssef et al.^2^ reviewed the various modifications of bubble columns such as sieve trays, structured packing, or vertical shafts, in combination with static mixers. All these modifications are suggested to reduce gas/liquid back-mixing and achieve a uniform bubble distribution, providing a better conversion yield. Out of these, Kiwi Miniskar et al.^3^ performed experiments with a microstructured bubble column (MSBC), i.e, a bubble column with trays of fibrous catalyst material. These studies provide design and modeling information for multistaged bubble columns, with and without reaction. Höller et al.^4^ studied the hydrodynamics in a nonreactive system by considering the effect of superficial gas velocity on various flow regimes. In a later study,^5^ the observed mass transfer coefficient was reported to be 10 times higher for a column with stages than that of a column without stages. Meikap et al.^6^ used a multistaged vertical cylindrical bubble column made of perspex fitted with a total of five disks with perforations (3 contraction disks and 2 expansion disks). The introduction of the hollow disks increases the mass transfer rate and gas holdup due to a higher interfacial area^7,8^ caused by bubble breakup and turbulence. Ito et al.^9^ also concluded the positive effect of the wires on bubble dynamics and in turn the reaction output in the column. Yang et al.^10^ analyzed the interaction between rising bubbles and sieve trays, to determine the bubble size distribution and the bubble breakup frequency. They reported that the main effect of a sieve tray is the added drag force and bubble breakup depending on the sieve pore size. Bubble breakup occurred when the sieve pore size was larger than the Sauter mean diameter; otherwise, the bubbles were slowed down. Sujatha et al.^11^ studied a micro-structured bubble column (MSBC) and found an increase in mass transfer coefficient for higher superficial gas velocities, due to increased bubble cutting and breakup. Chen et al.^12^ observed a similar bubble size reduction but a reduced cutting for liquids of higher viscosity. So, it can be concluded that knowledge of the effect of wire meshes and their interaction with gas bubbles and the effect on conversion/yield is important, which however is extremely complex and yet unpredictable. Baltussen^13^ used direct numerical simulations (DNS) to study the effect of bubble properties and wire mesh parameters such as Eötvös number and grid spacing on bubble passage/cutting. It was observed that larger bubbles (*Eo* > 4) could pass through, while smaller bubbles (*Eo* < 4) could only pass when the mesh spacing *s* was less than 0.625 times the bubble diameter. Wang et al.^14^ performed DNS simulations of two bubbles of different volumes impacting a cylindrical wire and observed that the bubbles do not come in direct contact with the cylinder. Instead, a separating liquid film is formed which eliminates any effect of surface wettability. This suggests that bubble cutting is independent of wire-surface properties. For larger-scale simulations, effective models are needed to account for this bubble–mesh interaction, including bubble passage and cutting. Jain et al.^15^ applied a simple geometrical cutting model to the Euler–Lagrangian simulations, in which the bubble is sectioned into several daughter bubbles depending on how much of the mother bubble is exposed to the individual grid openings. This model works fairly well; however, it lacks a physical basis.

In this paper, we analyze situations of a bubble passing a single mesh opening based on energy balance, and verify the model using the DNS results by Baltussen.^13^ The derived energy balance is then generalized to realistic conditions, i.e, large meshes with multiple openings in liquids of high and low viscosity. A new set of conditions describing the bubble fate (passage, cutting, or getting stuck) is obtained. These conditions can be readily used in Euler–Lagrangian simulations of microstructured bubble columns.

## Numerical Analysis of Bubble–Wire Interaction

When bubbles encounter a wire mesh, there are three possible outcomes: (i) they get cut into *n* > 1 daughter bubbles (*n* is the number of bubbles resulting from the mesh), (ii) they pass through by squeezing through an opening (*n* = 1), or (iii) they get stuck forming a gas layer behind the wire mesh (*n* = 0). For some flow conditions especially at higher superficial gas velocities, the daughter bubbles formed after cutting might immediately recoalesce.^11^ To be able to predict the outcome, we need to better understand the interaction between bubbles and wire meshes. These interactions are complex phenomena that involve competition of surface tension, viscosity, and buoyancy. To understand such interactions, we begin with a theoretical analysis using an energy balance of a bubble interacting with a wire mesh.

In our analysis, we make the following assumptions:

- The bubble comes to a rest after encountering the mesh. Such an assumption is reasonable as the bubble experiences increased drag force near the mesh, as it needs to satisfy the no-slip condition near the wire surface.
- The only possible bubble motion is sliding of the bubble interface along the wires.
- Effects of kinetic energy and energy loss due to drag can be neglected close to the mesh due to the lower velocity.
- We only consider the vertical motion of the bubble.

Consider a bubble of equivalent diameter *d*_*b*_ (major axis radius *a*, minor axis radius *b*) approaching a square mesh of wires of diameter *d*_*w*_, and skin-to-skin distance between the wires *s*, at an initial vertical speed of *u*_*y*_ (see Figure 2). The bubble experiences multiple contact zones when it touches the mesh depending on the scenario. Wang et al.^14^ observed through DNS studies that the presence of the liquid film that separates the gas from the cylinder surface effectively eliminates the influence of surface wettability on the bubble cutting process. Parts of the bubble that do not experience pressure force from the mesh are kept together with the help of surface tension. For the bubble to either pass or get cut, the buoyant energy has to exceed the surface energy. In order to find when this is the case, we analyze the different energy contributions of the bubble to obtain conditions for bubble passage.

**Figure 1:**
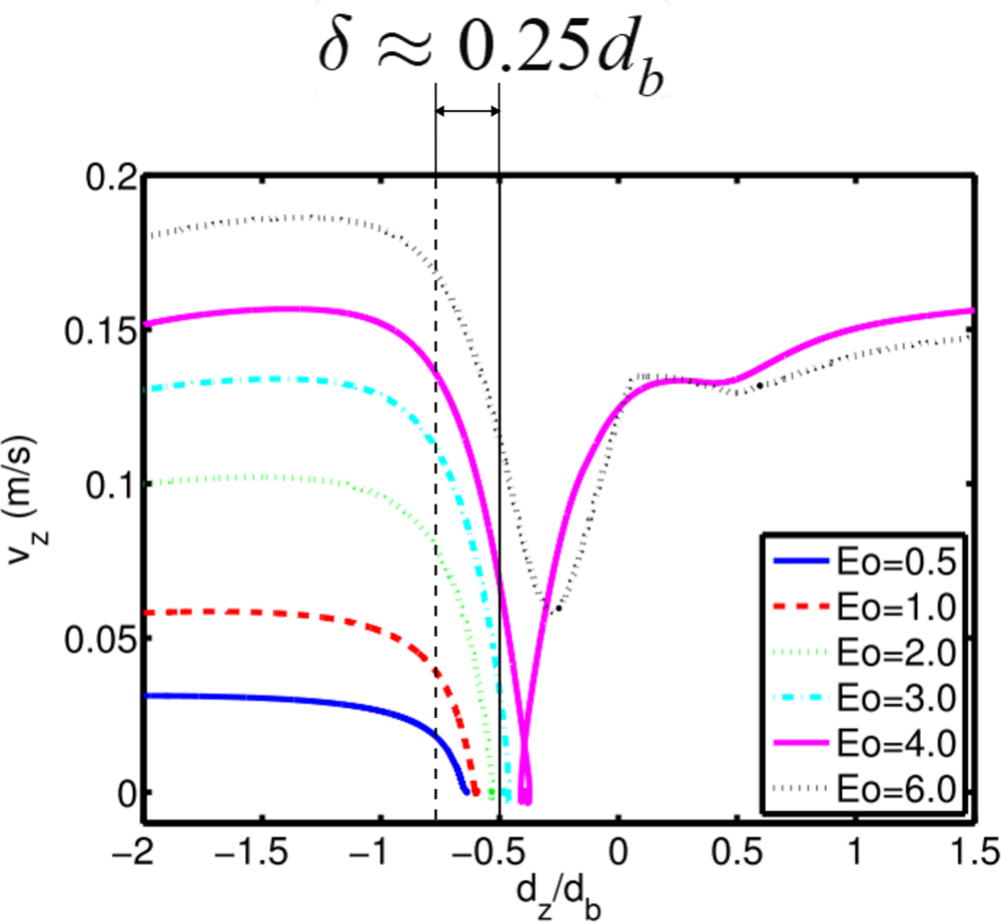
Calculation of δ using the DNS results by Baltussen;^13^ Velocity profiles of a bubble interacting with a single wire at different normalized distances between the bubble and the wire, *d*_*z*_/*d*_*b*_. Figure reproduced with permission.

The energy balance on a bubble near a wire mesh can be written as follows.
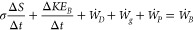
1The left-hand terms are the rate of change of surface energy () during the passage/cutting, rate of change of kinetic energy of the bubble (), work done by drag (*Ẇ*_*D*_), gravity work (*Ẇ*_*g*_), and pressure work by the wire (*Ẇ*_*P*_). The right-hand term is the work done by buoyancy (*Ẇ*_*B*_). Pressure work done by the wire mesh is considered zero as the points in proximity to the mesh are stationary (∫∇*p*_*w*_ ·**u** = 0). Gravity work done on the bubble is neglected (ρ_*g*_≪ ρ_*l*_, *W*_*g*_ ≈ 0). We can rearrange the equation as:
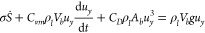
2where *C*_*vm*_ is the virtual mass, *C*_*D*_ drag coefficient, *V*_*b*_ the volume, and *A*_*b*_ the area of the bubble. Dividing eq 2 by the buoyancy work (input, ρ_*l*_*V*_*b*_*gu*_*y*_) and rearranging, we obtain a nondimensional equation.
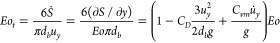
3where . Equation 3 shows that the change in dimensionless surface energy (LHS) balances the change in kinetic energy plus the work due to drag (RHS). The liquid sheet around the bubble decelerates near the wire mesh, thus offering assistance in bubble-cutting (*u̇*_*y*_ < 0). Even though the virtual mass term is crucial, its calculation is complex due to its transient nature. Work due to drag is close to zero as the velocity of the bubble near the wire-mesh is generally small (*u*_*y*_^2^ ≈ 0). After neglecting work due to drag (**Ẇ̇**_*D*_), eq 3 can be rewritten as:
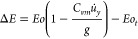
4where *ΔE* is the excess dimensionless energy for cutting/passage. The first two terms on the right-hand side are buoyancy (*Eo*) and virtual mass (), while *Eo*_*t*_ is the change in surface energy associated with the maximum surface deformation, which is expressed in a more practical form as:
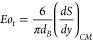
The contribution due to virtual mass is expressed as:
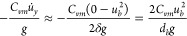
5where *u̇*_*y*_ is approximated as a constant value over a distance of δ, which is δ ≈ 0.25 *d*_*b*_ for the cases with liquid viscosity of 80 mPas as observed in the work of Baltussen^13^ and illustrated in Figure 1. After considering all the terms in eq 3, we can summarize that if *ΔE* > = 0, there is excess buoyant/kinetic energy as the bubble passes; whereas if *ΔE* < 0 there is too little energy and the bubble gets stuck. In the next paragraph, we introduce the different variables that can influence the cutting/passage process.

**Figure 2:**
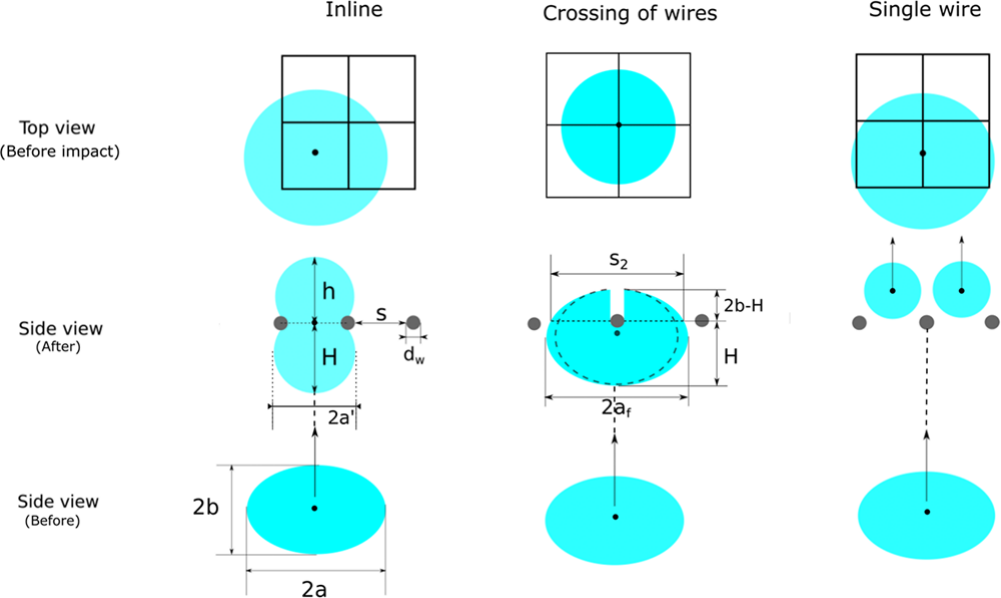
Schematic overview of the different parameters and scenarios of a bubble interacting with a wire mesh. Different bubble impact conditions produce different numbers of daughter bubbles for a bubble with the same diameter. The dots in the middle of the bubbles indicate the center of mass.

**Figure 3:**
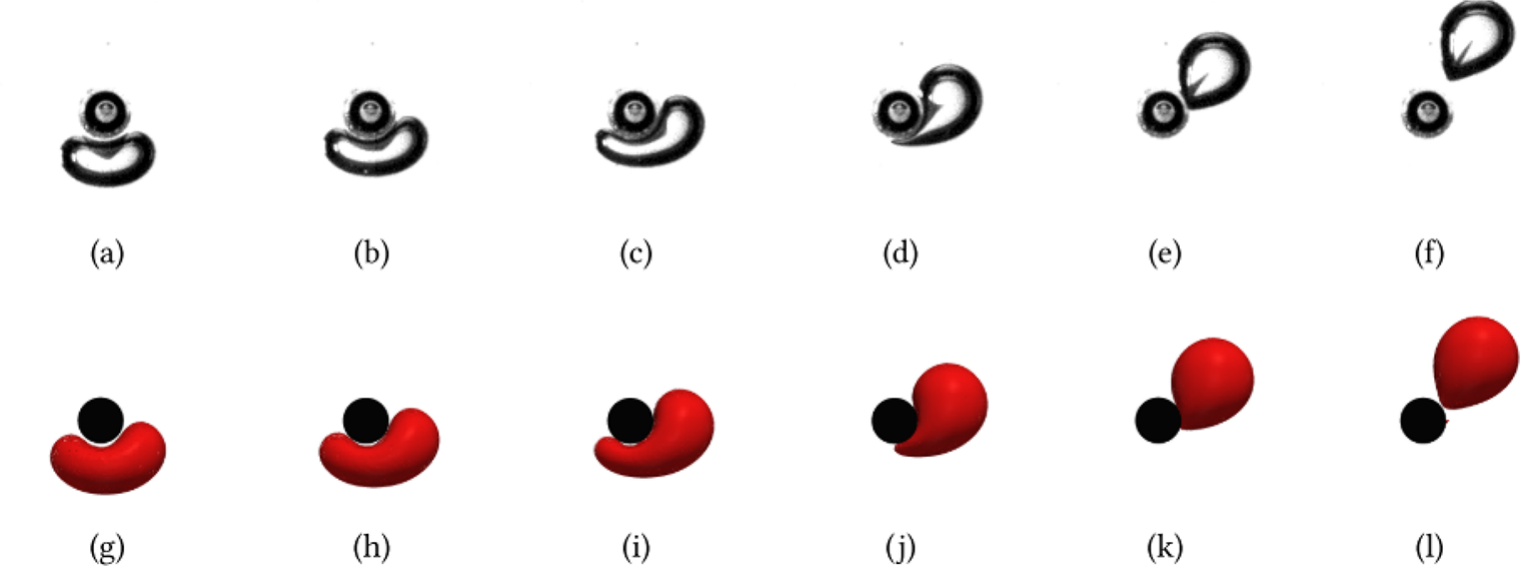
Horizontal shift of bubble in a highly viscous liquid (μ_*l*_ = 1.13 Pa.s). Experiments (a–f) and DNS results (g–l) reproduced with permission.^13^

The deformation barrier roughly increases with an increasing number of bubbles produced (*n*) as the surface energy increases substantially. Parameters such as the diameter to grid spacing ratio (*d*_*b*_/*s*) and the approach configuration (Figure 2) influence *n*, thus impacting the deformation barrier as well. In addition to that, it is more difficult for the bubble to pass through a smaller opening (*d*_*b*_/*s* ≫ 1). Although we have not included the effect of liquid viscosity (μ_*l*_) in the energy balance, viscosity plays an indirect, though an important role. When a bubble traveling through a viscous fluid hits a wire slightly off-center, the Laplace pressure difference between the two unequal bubble parts shifts the position of the bubble to the side with the least surface energy state as shown in Figure 3. If the time taken for the bubble to cut or pass (*t*_*c*_) is higher than the time taken for bubble drainage due to the Laplace differential pressure (*t*_*lp*_), the bubble can shift completely. In this way, viscosity plays an important role in hampering bubble cutting. In conclusion, we may consider the expression for the deformation barrier as:
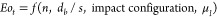
6

## Role of Viscosity

As discussed above, viscosity plays an indirect role in shifting the bubble approach conditions. This can be characterized by comparing the cutting (*t*_*c*_) and Laplace pressure driven drainage (*t*_*lp*_) time scales. We consider two limiting cases of bubble cutting i.e., in liquids of high (*t*_*c*_ ≫ *t*_*lp*_) and low viscosity (*t*_*c*_ ≪ *t*_*lp*_). At high viscosities, the viscous motion is slow and gives sufficient time for the bubble to accommodate itself to the lowest surface energy (i.e., by moving to an inline position). While for low viscosity, the bubble does not have enough time to shift to the lowest surface energy state.

### Low Viscosity

Once a bubble traveling through a low viscosity liquid (e.g., in gas–water system) encounters a wire mesh, it remains in its lateral position as it does not have enough time to realign to the position with the lowest surface energy. As the approach position is arbitrary, we reduce the complexity of the problem by restricting to three characteristic scenarios as illustrated in Figure 2. In these cases the center of mass of the bubble coincides with:

(i) the center of gap opening (“inline”),
(ii) the crossing of two wires (“crossing”), or
(iii) the middle of one of the wires (“single wire”).

#### Inline Configuration

First, we test our theory by comparing it with the inline case of Baltussen^13^ and extending it to larger meshes. Let us begin by defining the initial bubble as a spheroid of axes lengths *a* and *b*. The volume of the spheroidal bubble is expressed as . During a passage process, a spherical cap of height *h* forms above the wire mesh leaving behind a spheroidal mother cap of new axes lengths (*a*′ and *b*′), base length *s* (same as grid spacing), and height *H* as shown in Figure 2. The value of *H* is varied from 2*b* – *d*_*i*_ to *d*_*i*_, where *d*_*i*_ is the vertical displacement of the bubble contacting the wire with respect to the wire mesh. In order to calculate the total surface deformation rate, we use volume conservation to obtain the value of *h*. The total volume (mother + daughter cap) is expressed as:
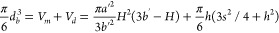
7where *V*_*m*_, *V*_*d*_ are the volume of the mother bubble (spheroidal) cap and daughter bubble (spherical) cap. Using an ellipse equation, *a*′ and *b*′ of the mother bubble can be related to *s* and *H*.
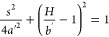
8During the passage process, the bubble deforms to fit in the grid spacing, thus gradually altering the axes length. Along with this, the eccentricity of the mother bubble cap varies with its volume; in general it will become more spherical with a higher aspect ratio *E*. The aspect ratio is estimated by using the correlation by Wellek et al.^16^ for the volume *V*_*m*_.
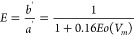
9

**Figure 4:**
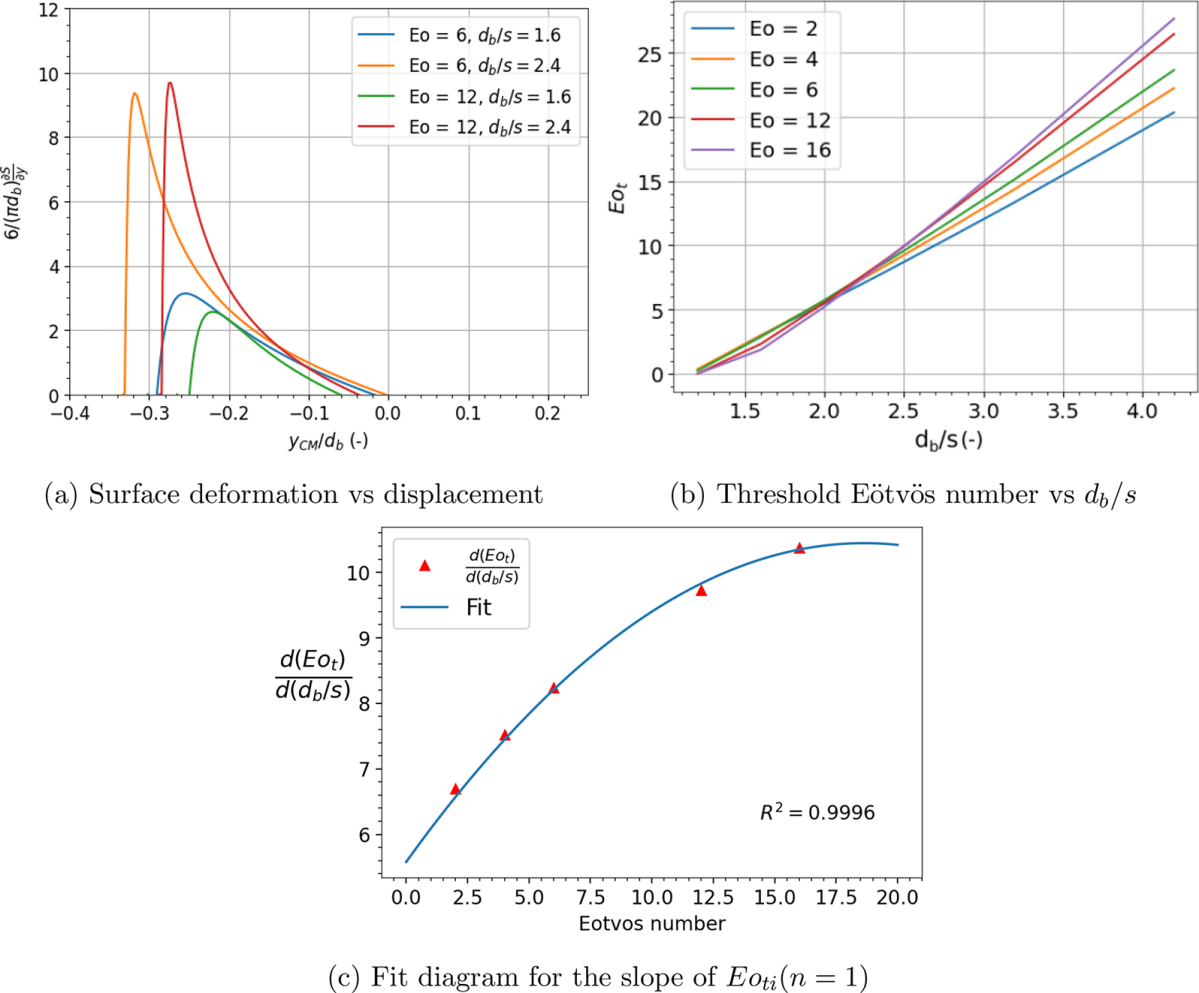
Deformation data such as (a) surface deformation, (b) threshold Eötvös number, and (c) fit diagram for bubble passage at inline configuration (*n* = 1).

**Figure 5:**
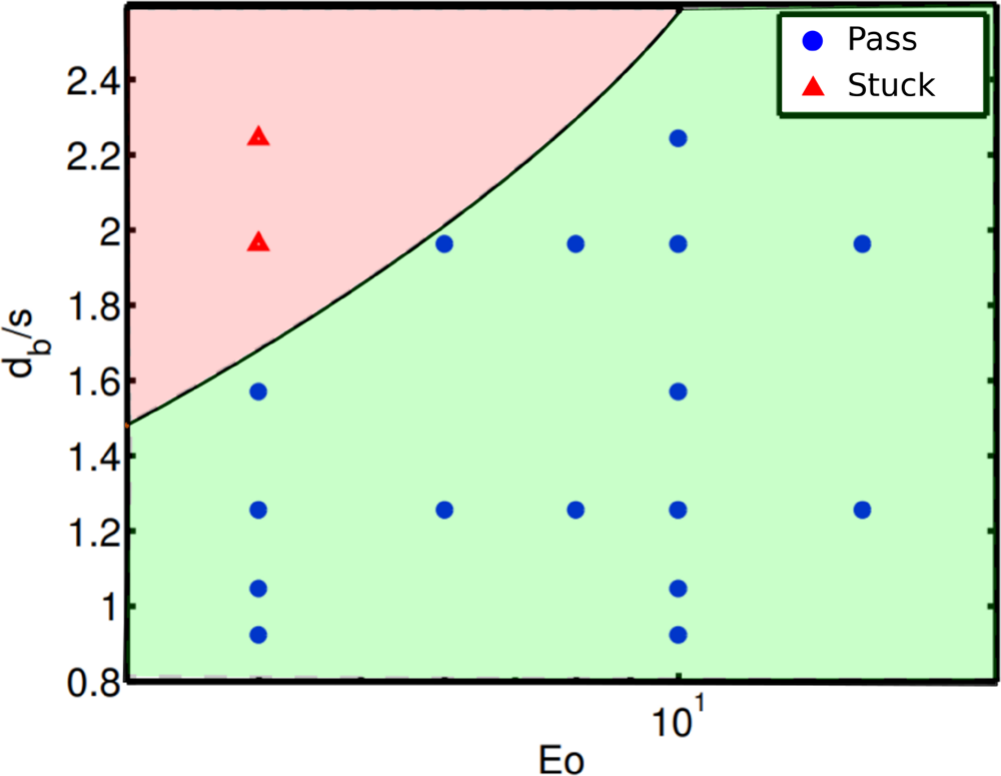
Regime map of the fate of a single bubble interacting inline with a wire mesh; Symbols represent the DNS data,^13^ while the solid line represents our results (eq 11).

Equations 8 and 9 are numerically solved in order to calculate *a*′ and *b*′ using successive substitution in small steps of *H*. These values are substituted in the cubic eq 7 to calculate *h*. The total surface area (mother + daughter cap) is calculated using the following equation:
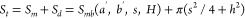
10where *S*_*mb*_ is the surface area of the mother bubble, which is calculated using a simple Python script. Equation 10 is differentiated with respect to the displacement to obtain its dimensionless form, 6(*dS*/*dy*_*CM*_)_*max*_/*πd*_*b*_, and plotted in Figure 4a. Figure 4a shows that the bubble starts to deform at different *y*_*CM*_/*d*_*b*_ values depending on both grid spacing (*d*_*b*_/*s*) and bubble diameter (*Eo*). In other words, the curve begins at different *y*_*CM*_/*d*_*b*_ values in the figure. The peak values of the curve are obtained and plotted for different *d*_*b*_/*s* values and Eötvös numbers in Figure 4. Grid spacing has more impact on the deformation barrier (*Eo*_*t*_) than the Eötvös number. Bubbles of higher Eötvös number encounter a slightly higher deformation barrier (*Eo*_*t*_) at higher grid spacing ratios (*d*_*b*_/*s* > 2.0). For the inline passage configuration, *Eo*_*ti*_ is written as:
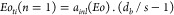
11where *a*_*inl*_ (*Eo*) = −0.014*Eo*^2^ + 0.522*Eo* + 5.58 is obtained by fitting the slopes of the curves in Figure 4 using a quadratic equation. The obtained threshold value *Eo*_*ti*_(*n* = 1) is compared with the regime diagram obtained by Baltussen^13^ in Figure 5. The threshold values accurately fall in the transition area between passage to stuck states obtained by Baltussen.^13^ Generally speaking the bubble passage becomes harder (i.e., higher Eotvos numbers can pass through) with increasing diameter-to-grid ratio (*d*_*b*_/*s*). Note that the virtual mass term is neglected, as both cases by Baltussen^13^ use liquids of high viscosity.

With this observation in hand, we extend the energy analysis to cutting (*n* > 1). For a bubble impacting an arbitrary *m* × *m* mesh (where *m* is an odd number) in an inline approach, we apply the same analysis procedure as done before. We assume that the number of daughter bubbles produced in this case is *m*^2^ and their heights are the same to reduce complexity. After the calculation of the total surface area of the composite bubble (mother + daughter bubbles), the associated deformation barrier (*Eo*_*t*_) is calculated and plotted for different *n*. We begin with the case of a bubble passing through a grid of *n* = 3 × 3 = 9. If all the daughter caps have a height of *h*, the total surface area is calculated as:
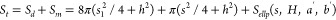
12in which *s*_1_ is the base diameter of 8 outer daughter bubbles.

**Figure 6:**
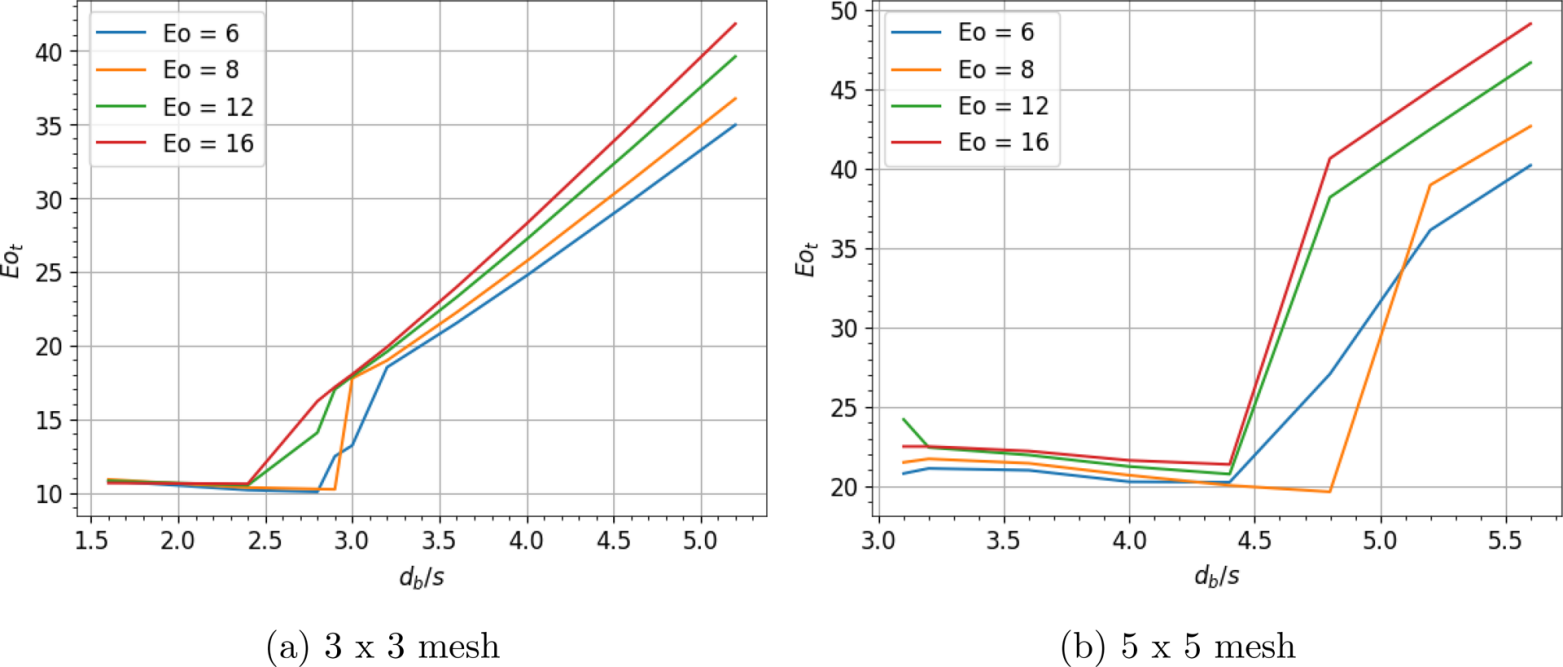
Deformation barrier for bubble cutting through (a) 3 × 3 mesh and (b) 5 × 5 mesh in an inline approach.

The new axes lengths *a*′ and *b*′ are calculated for a spheroid with its entry as explained in previous subsection. After obtaining the value of total surface area, it is differentiated with displacement to calculate the deformation barrier (*Eo*_*t*_) and the results are shown in Figure 6. The deformation barrier (*Eo*_*t*_) depends chiefly on *d*_*b*_/*s* but only slightly on the Eötvös number. For bubbles of higher Eötvös number, cutting is slightly harder. *Eo*_*t*_ stays relatively constant until a certain *d*_*b*_/*s* ≈ 2.6, after which it increases linearly. The transition region spans over a small range of *d*_*b*_/*s* values (2.5 < *d*_*b*_/*s* < 3) and is also modeled as a linear expression. The linear part is very close to *Eo*_*ti*_(*n* = 1) . Thus, we approximate the expression for *Eo*_*ti*_(*n* = 9) as:
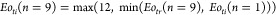
13where *Eo*_*tr*_(*n* = 9) = 12 + 6·(*d*_*b*_/*s* – 2.5) is the transition curve. The above-mentioned procedure for calculating *Eo*_*t*_ is also repeated for a grid producing 5 × 5 = 25 bubbles. With the number of daughter bubbles increasing, *Eo*_*t*_ increases as expected. In Figure 6b we observe that *Eo*_*t*_ for *n* = 25 consists of two limiting trends. For low *d*_*b*_/*s* values, *Eo*_*t*_ stays constant at a certain plateau. While for higher *d*_*b*_/*s*, *Eo*_*t*_ increases linearly. In between, the transition is treated as a linear function. This behavior can be fit by an equation of the following form:
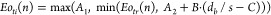
14where *A*_1_ is the plateau value, *A*_2_ is the *Eo*_*ti*_ value at which the linear part begins, *B* is the slope of the linear part, and *C* is the value of *d*_*b*_/*s* at the inflection point. All future plots that show similar trends (plateau + transition + linear) will be fitted in this form. Using this functional form for the fit, the threshold Eötvös number (*Eo*_*t*_) for *n* = 25 is approximated as:
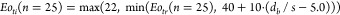
15where *Eo*_*tr*_(*n* = 25) = 22 + 36·(*d*_*b*_/*s*–4.5).

#### Crossing of Two Wires

When a bubble encounters the crossing of two wires, it requires more deformation compared to the inline configuration. This impedes bubble cutting in general. For a similar DNS case studied by Baltussen,^13^ the bubbles get split into 4 daughter bubbles through a mesh of 2 × 2. As the bubble passes through the wire mesh, a liquid-filled cavity is created inside the interior of the bubble above the two crossing wires. Because of volume conservation, the bubble is expanded and we assume that this expansion only takes place in the lateral direction, increasing the horizontal radius from *a* to *a*_*f*_. The value of *a*_*f*_ can be computed from a simple volume balance:
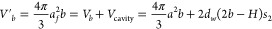
16where the cavity is approximated as two cuboids, each with dimensions *d*_*w*_ × (2*b* – *H*) × *s*_2_. Finally, this equation can easily be rewritten to obtain the value of *a*_*f*_.

**Figure 7:**
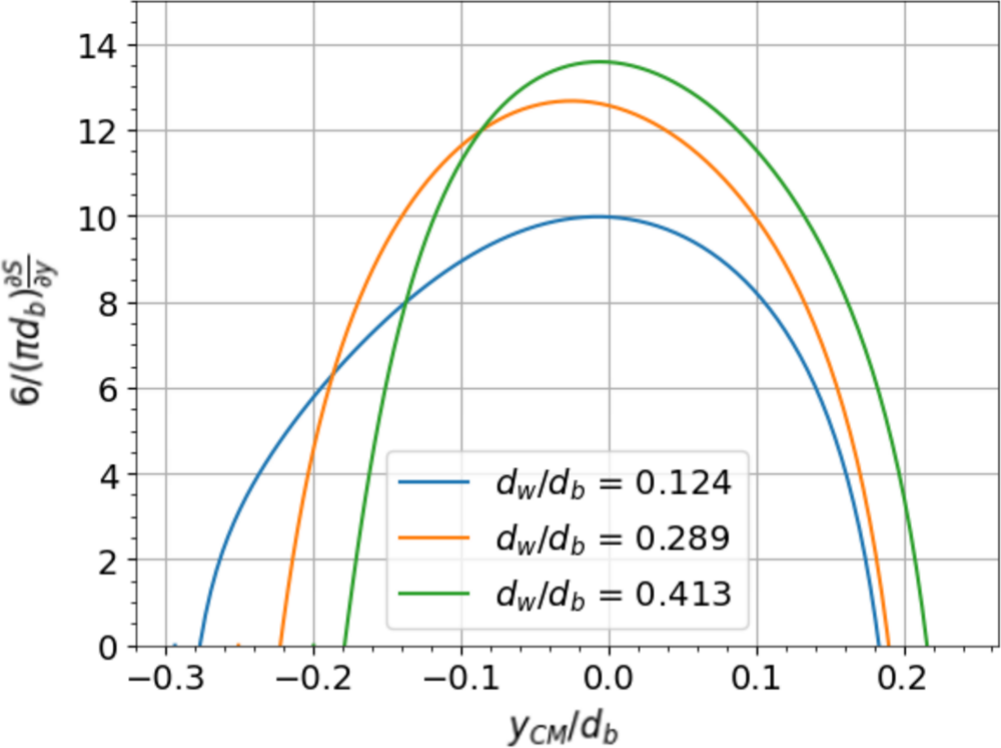
Effect of wire diameter on bubble deformation rate for a constant value of *d*_*b*_/*s* = 1.3.

**Figure 8:**
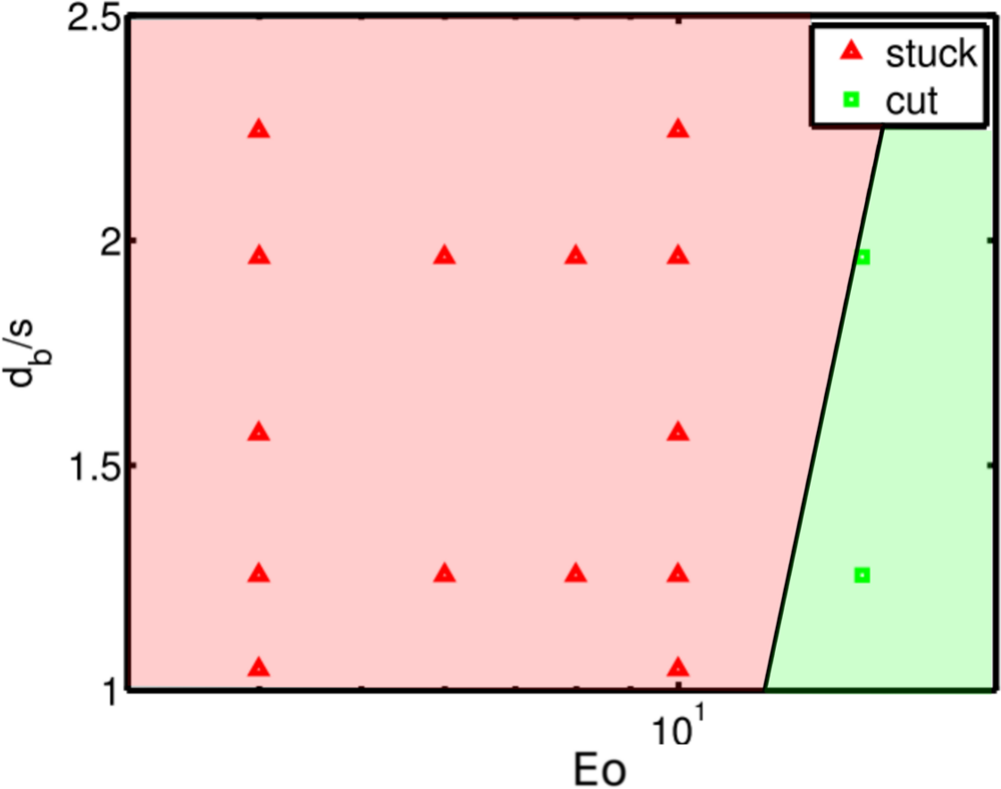
Regime map of the fate of a single bubble interacting with crossing of wires; the symbols represent the numerical data,^13^ while the solid line represents the values obtained from our analysis (eq 18).

The total surface area of the deformed bubble during the cutting process (*n* = 4) is expressed as:
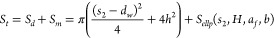
17where *s*_2_ is the lateral diameter of the daughter bubbles and *S*_*ellp*_ is the surface area of the mother bubble. Figure 7 shows the deformation rates against the center of mass of the bubble for *d*_*b*_/*s* = 1.3 and different wire diameter ratios (*d*_*w*_/*d*_*b*_ = 0.124, 0.289, 0.413), as well as its effects on the threshold Eötvös number. As expected, thicker wires impede bubble cutting, as the bubble has to deform more around the wire to cut. For the case of Baltussen,^13^*d*_*w*_/*d*_*b*_ takes a value of 0.32 and *Eo*_*t*_ is around 12.5. This value is correctly situated between the transition of the stuck to cutting regime observed in Figure 8. *Eo*_*t*_ for the same *d*_*w*_/*d*_*b*_ = 0.124 and other *d*_*b*_/*s* values are also obtained and plotted in Figure 8. A close match is observed with the DNS results.

**Figure 9:**
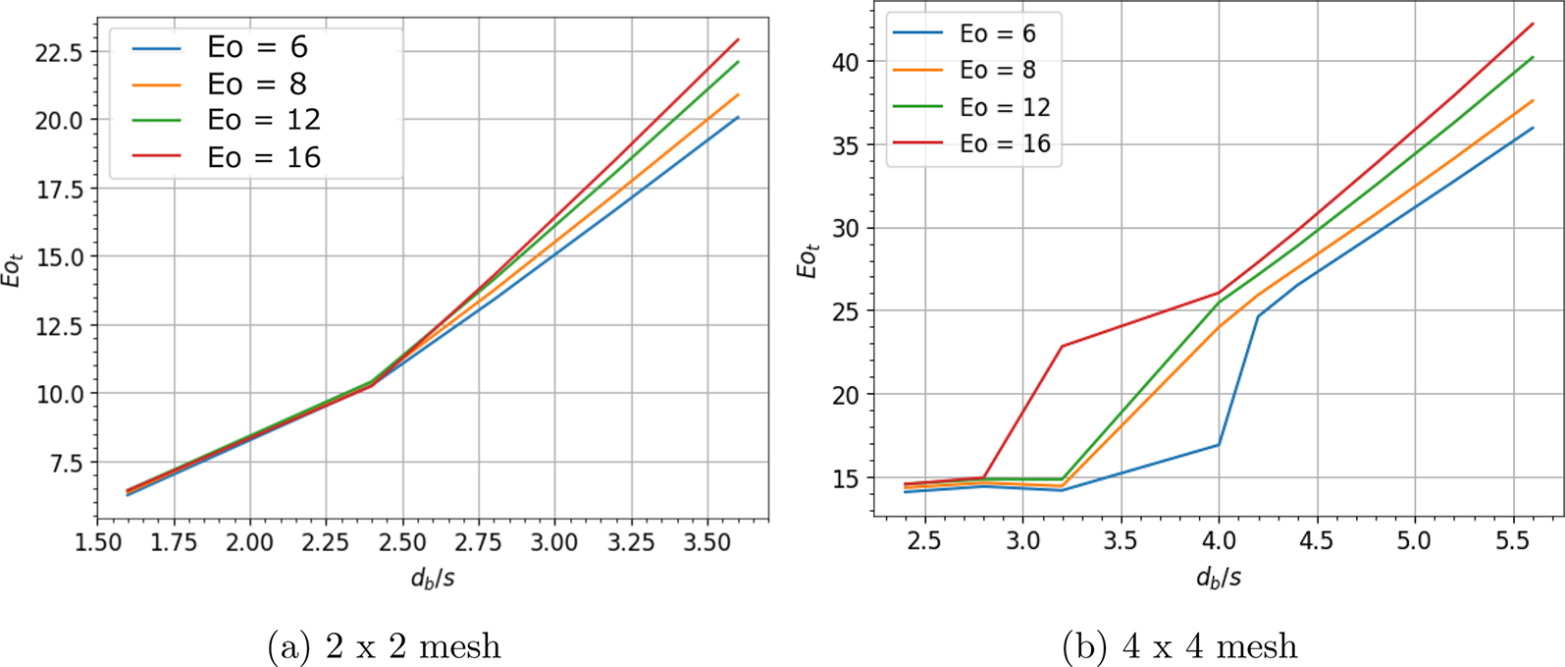
Deformation barrier for bubble cutting through (a) 2 × 2 mesh and (b) 4 × 4 mesh for *d*_*w*_/*d*_*b*_ = 0.

For the bubble cutting into 2 × 2 daughter bubbles with *d*_*w*_ ≈ 0, the threshold Eötvös number is simplified as:
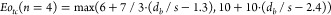
18For further analysis, we assume that the wire diameter is zero to simplify the calculations. Our analysis is extended to a crossing of 4 × 4 wires. The number of bubbles resulting from this wire mesh is considered to be 16, as it simplifies our study. For this case, the deformation barrier(*Eo*_*t*_) is plotted with *d*_*b*_/*s* in Figure 9. *Eo*_*tc*_ is expressed as follows:
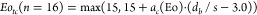
19and *a*_*c*_ = −0.003*Eo*^2^ + 0.313*Eo* + 6.067. The transition area is neglected in this case. It is observed that, for the same range of diameter to grid ratios, cutting is harder compared to the inline configuration. This is due to the larger number of daughter bubbles produced for the same *d*_*b*_/*s* as shown in Figure 2.

#### Single Wire

In this configuration, the bubble impinges on one of the wires and produces *m*·(*m* – 1) bubbles, i.e. *n* = 2, 6, 12, .... The deformation barrier is moderate and situated in between the previously mentioned configurations (i.e., between inline and crossing of wires).

**Figure 10:**
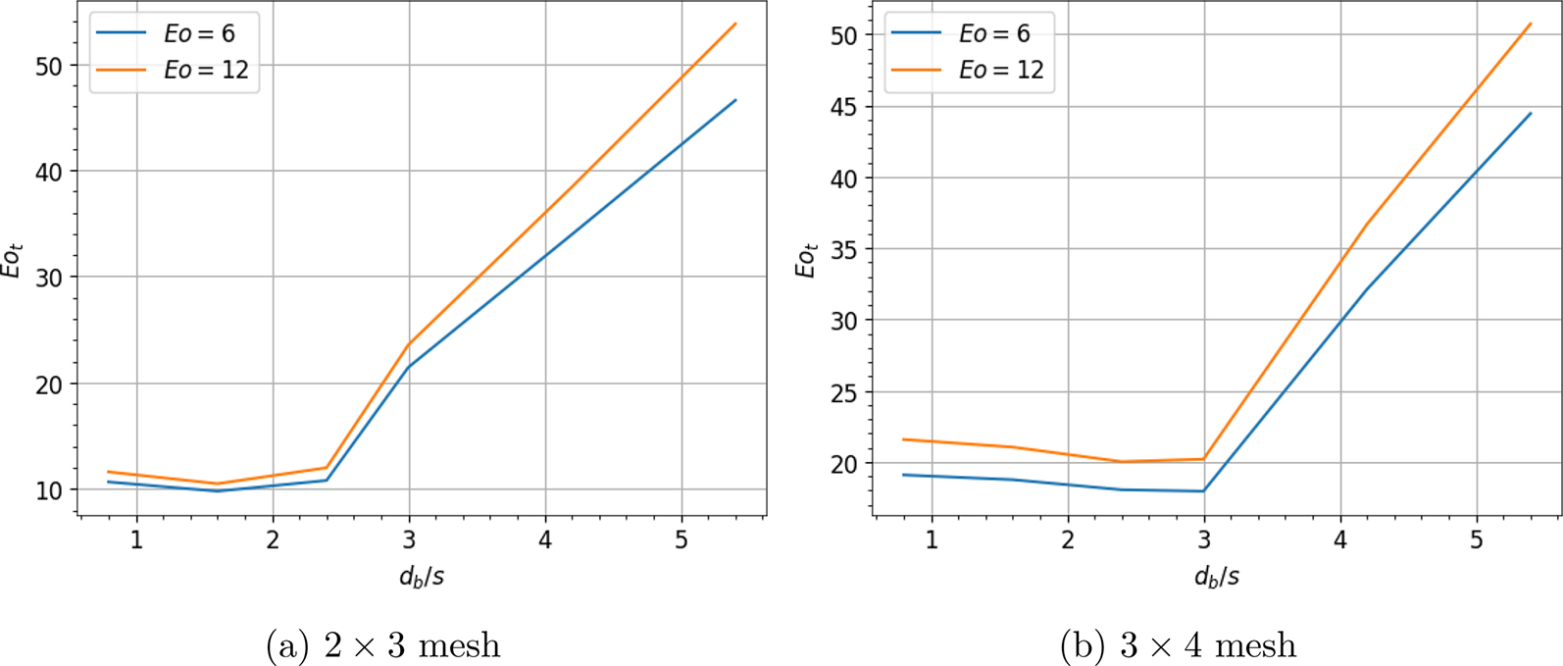
Deformation barrier for (a) 2 × 3 mesh and (b) 3 × 4 mesh with *d*_*b*_/*s*.

The bubble deformation rate is calculated as done before, for cases of a bubble passing through 2 × 3, 3 × 4, and 4 × 5 mesh openings and for different values of *d*_*b*_/*s*. The results are shown in Figure 10. As expected the barrier to cutting increases with the number of daughter bubbles. The expression for *Eo*_*to*_ of daughter bubbles (*n* = 2) is written in simplified form after fitting:
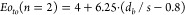
20Similarly *Eo*_*to*_ for *n* = 6, 12, 20 are written as:
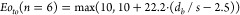
21
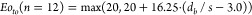
22
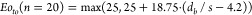
23

### High Viscosity

So far, we have only considered one limiting case, i.e. that of a low liquid viscosity, where we considered three different interaction scenarios. For liquids of high viscosity there is one important difference. At high viscosities, the bubble has enough time to realign to its lowest energy state. Thus, the bubble impacting in any configuration will reconfigure to the inline position, thus simplifying our calculation. Equations 11, 13, and 15 are used for calculating *Eo*_*t*_ for this case. In the next section, we will discuss how the expressions for the threshold Eötvös number can be used as a closure model for bubbly flow simulations.

## Closure Model for Bubble-Column Simulations

As found in the previous section, bubble cutting/passage depends on the number of daughter bubbles produced, the bubble impact configuration, *Eo*, and *d*_*b*_/*s*. The cases examined here are some typical well-defined scenarios, but bubbles can impact at any arbitrary position on a wire mesh. To simplify the whole cutting model, we apply a stochastic model to a more general case of cutting. A stochastic model for cutting is appropriate in Euler–Lagrangian simulations, in which bubble path prediction is not very well resolved. To account for the different possible scenarios, we introduce a probability for each of them. The probabilities for the bubble to impact inline, crossing, or one of the wires are indicated as *P*_*i*_, *P*_*c*_, and *P*_*o*_, respectively.

**Figure 11:**
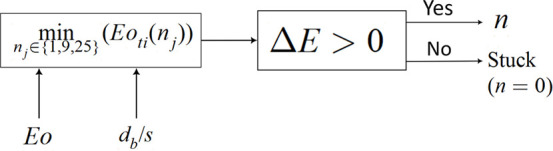
Block diagram of bubble cutting model for liquids with high viscosity.

For liquids with high viscosity, all bubbles tend to reach the inline position due to its high stability compared to the other two configurations. As a result, the probabilities for cutting are as follows: *P*_*i*_ = 1, *P*_*o*_ = 0, *P*_*c*_ = 0. In the inline configuration, the bubble takes the path with least necessary energy to cut. This path is found by taking the minimum of all possible thresholds (*Eo*_*ti*_ (*n*_*j*_)) for different daughter bubbles *n*_*j*_. The resultant threshold Eötvös number (*Eo*_*ti*_(*n*)) for inline configuration is thus obtained as:
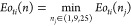
24where *n* is the number of daughter bubbles produced after the minimum operation, i.e., *n* = *n*_*j*_ corresponding to the minimum *Eo*_*ti*_ (*n*_*j*_) in the inline configuration. The necessary steps for inclusion in a closure model are drawn in the form of a block diagram in Figure 11. *Eo* and *d*_*b*_/*s* are the input quantities for the evaluation of minimum threshold Eötvös number for inline configuration (*Eo*_*ti*_), which is subsequently compared to the bubble Eötvös number. When *ΔE* is positive (i.e., *Eo* > *Eo*_*ti*_), the bubble passes through or is cut into *n* daughter bubbles.

**Figure 12:**
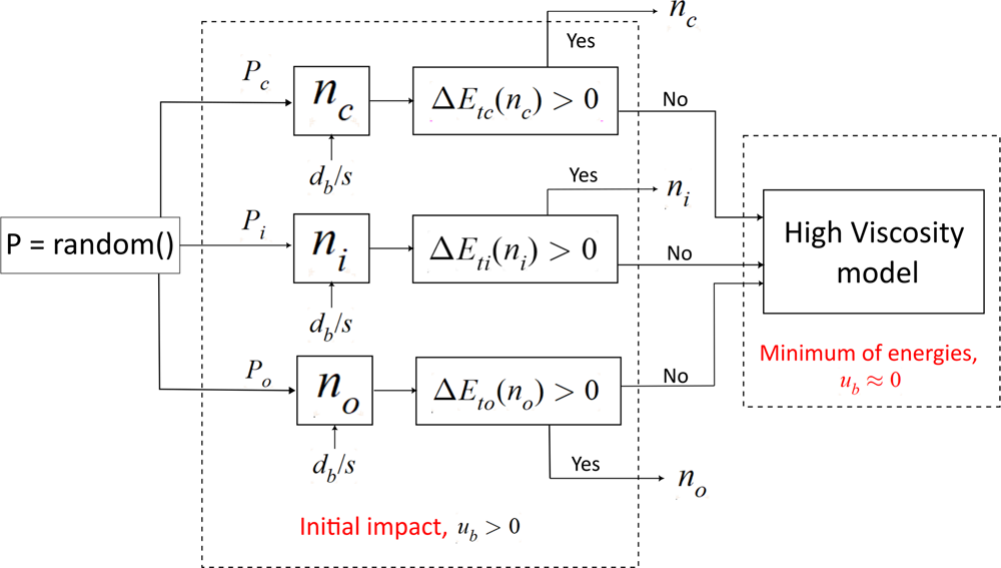
Block diagram of bubble cutting model for liquids with low viscosity.

**Figure 13:**
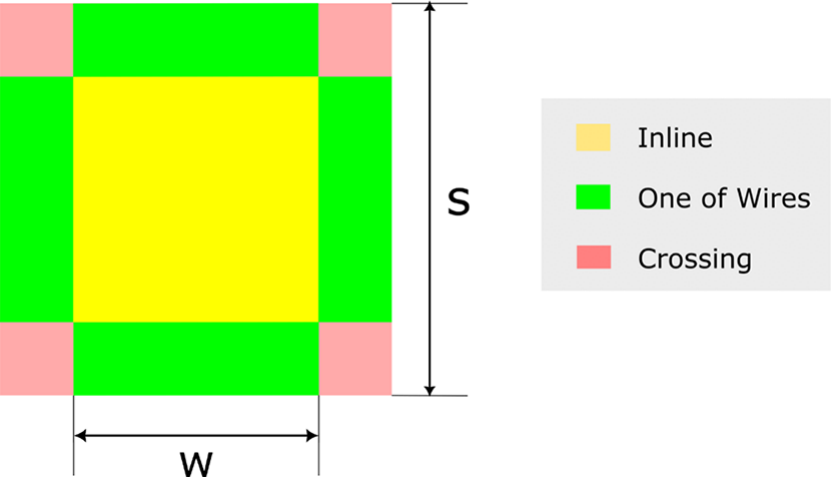
Different configurations in a mesh.

As for low-viscosity fluids, e.g., in the air–water system, bubble cutting is proposed to involve more steps than in high-viscosity fluid systems (Figure 12). This is because the bubbles do not have enough time to realign to its lowest surface energy state, when interacting with the wire mesh. The three configurations, i.e., inline, crossing, and single wire, are the chief scenarios applied in this case. However, in reality, bubbles can be at an arbitrary position with respect to the wire mesh. Thus, we need to represent the arbitrary case by expanding the tolerance for the definitions of the three chief configurations. For example, the inline position refers to the case where the bubble’s center of mass aligns with the grid opening. Alternatively, it is considered to be the case where the number of mesh openings covered by the bubble is *m*^2^, in which *m* is an odd integer (assuming a square bubble projection area with side *d*_*b*_). This definition provides us with an approximate area where the bubble center approaches inline (see Figure 13). As a result, we define the probabilities of the three chief configurations based on the location of bubble mass center relative to the center of a mesh opening:
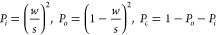
25And *w*, as the width of inline configuration, is defined as:
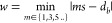
26

Similar to Figure 11 for high-viscosity fluid systems, the bubble cutting model for low-viscosity fluid system is sketched in Figure 12. Immediately after a bubble hits the wire mesh, a random number, *p* ∈ [0, 1], is picked using a generator. The ranges are associated with the outcomes. Depending on which corresponding range *p* lies, the outcome is chosen. For example, *P*_*c*_ = 0.1, *P*_*o*_ = 0.3, *P*_*i*_ = 0.6, and *p* = 0.5, and then the outcome is the inline configuration (0.4 < *p* < 1.0). The order of ranges is inconsequential as *p* is uniformly distributed. After the outcome is chosen, the number of daughter bubbles is calculated. The number of daughter bubbles produced for the inline (*n*_*i*_), crossing (*n*_*c*_), and single wire configuration (*n*_*o*_) is dependent on the grid openings that the bubble is exposed to in that configuration. Assuming a square projection area of the bubble with side *d*_*b*_, it is expressed as:
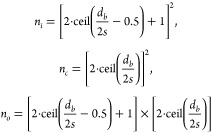
where ceil(*x*) is the ceiling function. *ΔE*_*tc*_(*n*_*c*_) and *ΔE*_*to*_(*n*_*o*_) are the excess energies for crossing and single-wire configurations. If the excess energy is positive, cutting occurs as expected. If there is insufficient energy for cutting, the bubble stops beneath the mesh and the virtual mass term depletes completely (*u*_*b*_ = 0). As a result, the bubble rearranges itself to a stable configuration, i.e., inline, while looking for the minimum energy state. From here, the same steps as in high-viscosity model are followed.

The bubble cutting model involves several steps. The above-mentioned mechanisms for cutting require further studies and validation. As a final remark, one shall keep in mind that the stochastic models presented in Figures 11 and 12 do not capture all the scenarios of bubble-mesh interactions in practice, but rather the chief configurations studied in this work.

## Conclusions

Using an energy balance, we obtained rules and expressions that provide the outcome of an interaction of a rising bubble with a wire mesh, for three typical configurations (inline, crossing, and single wire) at two viscous limits (high and low). Depending on the number of daughter bubbles produced, the obtained necessary energy, expressed as *Eo*_*t*_, shows a plateau until a certain grid spacing ratio (*d*_*b*_/*s*), after which it grows linearly. The energy threshold (*Eo*_*t*_) is found to increase with *d*_*b*_/*s* and *n*. The obtained results are verified with the DNS studies of Baltussen.^13^ Depending on the liquid viscosity limit (determined by the comparison of Laplace-pressure drainage time and cutting time) the bubble can either reconfigure to the lowest surface energy state or follow the default state. A closure model is created for different limiting cases by combining deformation barrier *Eo*_*t*_ from these cases. In future work, we will use this model in Euler–Lagrangian simulations of bubbly flows in microstructured bubble columns. Studies on moderate viscosity are necessary for a more generalized cutting model, which is beyond the scope of this work. In our work we neglected effects drag and virtual mass; including these may lead to a further refined model.

## Supporting Information Available

Appendix, Deformation barrier for bubble passing through a 3 × 3 mesh. (PDF)
