# Supplementary material for: A Model for the Fate of a Gas Bubble Interacting with a Wire Mesh
Source: Ind Eng Chem Res. 2023 May 8;62(45):19302–10. doi: 10.1021/acs.iecr.3c00265 (PMC10655082; doi:10.1021/acs.iecr.3c00265)
Supplement: Supplementary file 1 — ie3c00265_si_001.pdf [file ie3c00265_si_001.pdf]

Supporting Information

A model for the fate of a gas bubble interacting with a wire mesh

Rahul Subburaj[1], Yali Tang[1,2*], Niels G. Deen[1,2]

1. Power & Flow Group, Department of Mechanical Engineering, Eindhoven University of Technology, P.O. Box 513,
5600 MB, Eindhoven, The Netherlands

2. Eindhoven Institute for Renewable Energy Systems (EIRES), Eindhoven University of Technology, P.O. Box 513,
5600 MB, Eindhoven, The Netherlands

* Corresponding author – y.tang2@tue.nl

Deformation barrier for 3 x 3 mesh

We begin with the case of a bubble passing through a grid of $n = 3 \times 3 = 9$. If all the daughter caps have a height of h, the total surface area is calculated as:

$$S_t = S_d + S_m = 8\pi\left(\frac{s_1^2}{4} + h^2\right) + \pi\left(\frac{s^2}{4} + h^2\right) + S_{ellp}\,(s, H, a', b')$$

in which s_1 is the base diameter of 8 outer daughter bubbles. During cutting these bubbles does not necessarily fit into the mesh-opening, thus having a different value than s. In the case of a bubble cutting through 3 x 3 mesh, s_1 (width of the outer daughter bubble base diameter) can be calculated by:

$$s_1 = \frac{S - s}{2}$$

where S is the mother bubble base diameter. The new axes lengths a' and b' are calculated for an ellipsoid with its entry as explained in section 3.1i of the manuscript. After obtaining the value of total surface area, it is differentiated with displacement to calculate the deformation barrier (Eo_t).

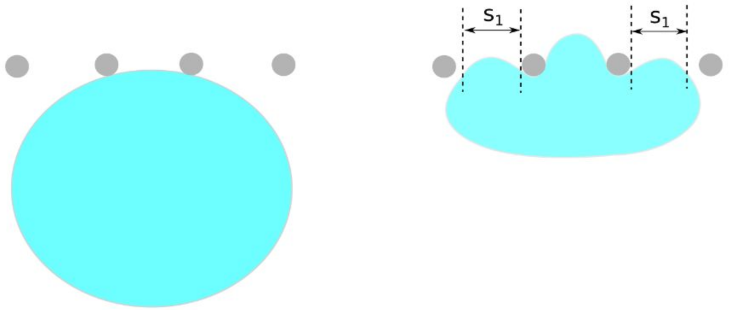

S1: Deformation of a bubble passing through a 3 x 3 mesh in inline fashion.
